# Efficacy and safety of traditional Chinese medicine in patients with acute exacerbation of idiopathic pulmonary fibrosis: study protocol for randomized, controlled, exploratory clinical trial

**DOI:** 10.1186/s13063-022-06026-0

**Published:** 2022-02-02

**Authors:** Zhang Hailong, Li Jiansheng, Guo Wen, Wang Lu, Zhang Dong, Zhao Limin, Zhou Miao, Luo Weixian

**Affiliations:** 1 Co-construction Collaborative Innovation Center for Chinese Medicine and Respiratory Diseases by Henan & Education Ministry of P.R. China, Henan Key Laboratory of Chinese Medicine for Respiratory Disease, Henan University of Chinese Medicine, Zhengzhou, China; 2 The First Affiliated Hospital of Henan University of Chinese Medicine, Zhengzhou, China; 3 Henan Provincial People’s Hospital, Zhengzhou, China; 4 The Third Affiliated Hospital of Henan University of Chinese Medicine, Zhengzhou, China; 5 Zhengzhou First People’s Hospital, Zhengzhou, China

**Keywords:** Idiopathic pulmonary fibrosis, Acute exacerbation, Traditional Chinese medicine, Randomized controlled trial, AE-IPF

## Abstract

**Background:**

At present, there is short of effective treatment for acute exacerbation of idiopathic pulmonary fibrosis (AE-IPF). The treatment of IPF with traditional Chinese medicine (TCM) has some advantages. However, the evidence is unclear whether TCM can be recommended as an effective therapy to treat AE-IPF. The purpose of the study is to explore the efficacy and safety of TCM for patients with AE-IPF.

**Methods:**

A randomized, double-blind, placebo-controlled, exploratory clinical trial will be performed. A total of 80 patients diagnosed with AE-IPF will be randomized into the intervention or control group. In addition to conventional treatment, the intervention group will be treated with Kangxianhuanji granule, and the control group will be given a placebo granule. The administration frequency is 10 g each time and two times daily. After 4 weeks of treatment, the patients were followed up for 12 weeks. The primary outcomes are treatment failure rate and all-cause mortality. Secondary outcome measures will include the length of hospitalization, overall survival, acute exacerbation rate, intubation rate, Modified British Medical Research Council (mMRC) score, the St George’s Respiratory Questionnaire idiopathic pulmonary fibrosis (SGRQ-I) score, and arterial blood gas analysis.

**Discussion:**

TCM may be beneficial in IPF. However, it has never been evaluated in patients with AE-IPF, who are incredibly prone to respiratory failure and have a high mortality rate. It is the first clinical trial to explore the efficacy and safety of TCM in the treatment of AE-IPF. This result will provide a basis for further study, which provides a high-quality evidence for the treatment of AE-IPF with TCM.

**Trial registration:**

Chinese Clinical Trial Registry ChiCTR1900026289. Registered on 29 September 2019.

## Background

Idiopathic pulmonary fibrosis (IPF) is a chronic, progressive, fibrotic lung disease of unknown etiology occurring in adults [1]. It is characterized by worsening lung function and leading to respiratory failure and death [2]. Post-diagnosis median survival of patients with IPF has been reported to range from 2 to 3 years [1, 3–6]. However, some IPF patients have experienced a rapid progression of respiratory failure, which is called acute exacerbation of IPF (AE-IPF). AE-IPF is one of the leading causes of deterioration and mortality of patients with IPF [7–9]. AE-IPF can occur at any stage of IPF, with a dangerous condition, rapid progress, high mortality, and poor prognosis. The incidence of AE-IPF reported in the literature was significantly different due to the lack of a unified diagnostic standard, research methods, and different populations. The annual incidence of IPF has recently been estimated at 5~10% in the official Clinical Practice Guideline [1]. In many prospective studies, the annual incidence rate was slightly higher than 20% [10–12]. According to reports, the median survival period is about 3–4 months, and the mortality rate during hospitalization after acute exacerbation is 55–80% [1, 11, 13]. Nearly 90% of patients with AE-IPF need to be treated in ICU [14], and those who survive after the first acute exacerbation have a mortality rate of more than 90% after 6 months [15]. Therefore, the prevention, early diagnosis, and active treatment of AE-IPF are the primary and arduous medical tasks in clinical practice. It is particularly important to carry out therapeutic research on AE-IPF.

Due to the critical condition, rapid progress, and poor prognosis of patients with AE-IPF, the research and understanding of AE-IPF have been deepened in recent years. However, the etiology and mechanism of occurrence are still unclear. AE-IPF is challenging to treat and has a poor prognosis. There are no clear and valid treatment measures and drugs. At present, hormones, anti-fibrosis, and anti-infection are mainly used, and the efficacy of these drugs is not accurate. There were some specific effects and advantages of IPF patients treated by traditional Chinese medicine (TCM) [16–18], but mostly focusing on the stable period. The treatment of AE-IPF with TCM is still in the exploratory stage. Based on the evidence of the potential advantages of TCM for IPF patients, we will perform the clinical pilot study investigating the efficacy and safety of TCM in the treatment of AE-IPF.

## Methods/design

### Objectives

This trial aims to explore the safety, efficacy, and feasibility of TCM (Kangxianhuanji granule) in patients with AE-IPF.

### Study design

**Fig. 1:**
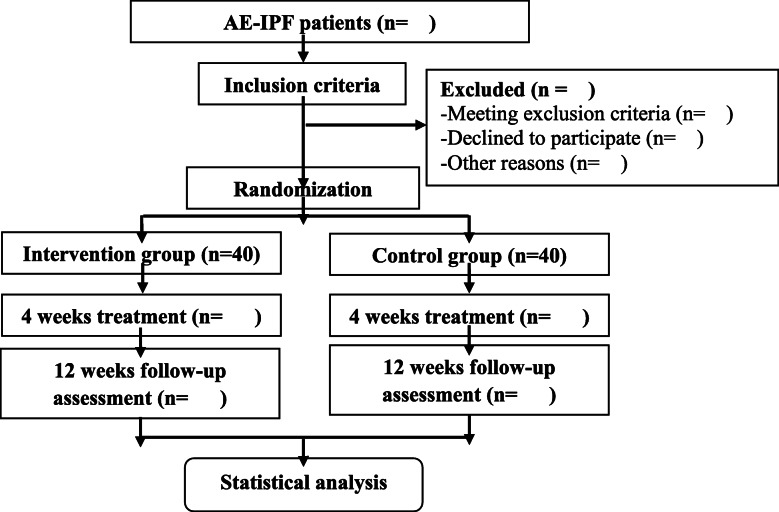
Flow chart for the intervention trial

This study is a randomized, double-blind, placebo-controlled exploratory clinical trial. After receiving written informed consent, the subjects who passed the screening criteria will be recruited and randomly divided into the intervention group or the control group at a ratio of 1:1. After 4 weeks of treatment, the patients were followed up for 12 weeks. The study flow chart is summarized in Fig. 1, and the timing of treatment and follow-up are detailed in Table 1.

**Table 1:** Timing of treatment visits and data collection

	Baseline	Treatment			Follow-up		
	Day 0	Day 7	Week 2	Week 4	Week 8	Week 12	Week 16
	V1	V2	V3	V4	V5	V6	V7
**Sign informed consent**	**×**						
**Randomization**	**×**						
**Medical history**	**×**						
**Intervention**	**×**	**×**	**×**				
**Primary outcome**							
Treatment failure rate		**×**	**×**	**×**			
All-cause mortality		**×**	**×**	**×**	**×**	**×**	**×**
**Secondary outcomes**							
Length of hospitalization		**×**	**×**	**×**			
Overall survival		**×**	**×**	**×**	**×**	**×**	**×**
Acute exacerbation rate					**×**	**×**	**×**
Intubation rate		**×**	**×**	**×**	**×**	**×**	**×**
mMRC	**×**	**×**	**×**	**×**			**×**
SGRQ-I	**×**	**×**	**×**	**×**			**×**
Arterial blood gas analysis	**×**	**×**	**×**	**×**			

Abbreviations: *mMRC* Modified British Medical Research Council, *SGRQ-I* The St George’s Respiratory Questionnaire for Idiopathic Pulmonary Fibrosis

The protocol of the trial (version 2019.06.18.1.0.4.0, 18 June 2019) was approved by the ethics committees of the First Affiliated Hospital of Henan University of Chinese Medicine (No. 2019HL-063-01) and registered at www.chictr.org.cn on September 29, 2019 (ChiCTR1900026289). The sponsor of the study is the Henan University of Chinese Medicine. The funder has no role in study design, data analysis, or the decision to submit the report for publication. In order to ensure the scientific nature of the trial, the reliability of the data, and the safety of the participants, the sponsor has established a Data and Safety Monitoring Boards (DSMB) which consists of medical, clinical pharmacology, epidemiology, statistics, and ethics experts. The protocol follows the recommendations of the SPIRIT initiative [19].

### Research centers

The study will be carried out in the respiratory ward of 4 hospitals in China. The name of the hospitals as follows:

1. The First Affiliated Hospital of Henan University of Chinese Medicine;
2. The Third Affiliated Hospital of Henan University of Chinese Medicine;
3. Henan Provincial People’s Hospital; and
4. Zhengzhou First People’s Hospital.

### Sample size

It is the first exploratory study to explore the efficacy of TCM in patients with AE-IPF. At present, we have not found the research data of AE-IPF treated by TCM, and the formal sample size calculation is not possible. We referred to the calculation method of the minimum sample size in exploratory clinical research, 40 participants in the intervention group, and 40 in the control group (*N* = 80 in total). The result of this study will provide data for a formal sample size calculation for further study.

### Participants

AE-IPF is defined as a clinically significant acute respiratory disease with worsening respiratory symptoms. It is characterized by a new and widespread alveolar abnormality on lung computed tomography [7]. The diagnosis of AE-IPF will be based on the Standards of An International Working Group Report [7]. To promote recruitment as well as compliance, a green channel for participants to see the doctor was established, regular phone calls or WeChat reminders of the follow-up date, provision of education manuals, and reduce or exempt medical expenses. For those who are lost to follow-up, try to get in touch through as many channels as possible. If the data is missing due to dropouts, we will deal with it through statistical methods, such as the intention-to-treat (ITT) analysis.

### Recruitment

The recruitment process of participants mainly includes recruiting participants, screening qualified participants, and obtaining informed consent of participants. The following is our process of recruiting participants. First, we determined that the doctors in each research center are responsible for recruiting staff. Second, we formulated recruitment plans and needs. Third, we carried out recruitment publicity through recruitment advertisements, which were placed at the entrance of the ward. Fourth, all participants would be recruited in the ward. Fifth, each research center was responsible for receiving visiting participants by special personnel, and at the same time, we should also answer the questions from online consultants. Sixth, we should screen the eligibility of participants according to the inclusion and exclusion criteria and doctors’ clinical experience. Finally, we should give a detailed description of the clinical trial scheme for the qualified participants, and inform them of the possible benefits and risks.

#### Inclusion criteria

1. A confirmed diagnosis of AE-IPF;
2. Age between 40 and 80 years;
3. Without participation in other interventional trials in 1 month; and
4. With the informed consent signed.

#### Exclusion criteria

1. Pregnant or lactating women;
2. Dementia, mental disorders, and reluctant partners;
3. Complicated with heart failure (NYHA Class IV), or unstable hemodynamics, or hypertension (Grade-4), or severe cardiac arrhythmias (e.g., ventricular tachycardia, paroxysmal supraventricular tachycardia, ventricular flutter, and ventricular fibrillation);
4. Complicated with other respiratory diseases (e.g., pulmonary thromboembolic, active tuberculosis, bronchiectasis, and pneumothorax);
5. Have been treated for more than 7 days before inclusion in the study;
6. Respiratory failure requiring invasive mechanical ventilation;
7. Complicated with severe liver and kidney disease (cirrhosis, portal hypertension, dialysis, or after renal transplantation);
8. Bedridden for various reasons; and
9. Allergic to the study of drugs.

#### Withdrawal, dropout, and discontinuation

1. During the study period, the disease deteriorated (such as complicated with acute respiratory failure, acute myocardial infarction, or acute heart failure, etc.);
2. Serious adverse events occurring in the study;
3. Serious violation of the research protocol makes it difficult to evaluate the effect of the drug, such as not taking medicine on time or not participating in the follow-up on time;
4. Unwilling to continue participating in clinical trials;
5. Lost participants during the study.

### Randomization and blinding

The multicenter, dynamic, blocked randomization method is adopted. An independent statistician will produce the random allocation sequence via the PLAN sentences of the Strategic Applications Software (SAS®) Version 9.2 (KEY: FQ37-WSB8-7G5C). After signing the informed consent, the eligible participants will be allocated randomly into either the intervention group or control group at a 1:1 ratio using the SAS9.2 software with a block size of 4. The scheme of random allocation will be obtained from a central stochastic distribution system provided by Jiangsu Famous Medical Technology Co., Ltd. in Nanjing, China.

Except for those responsible for blinding, all participants and investigators will be blinded to the group. The odor and color of Kangxianhuanji granule and placebo look the same, and the packaging is the same. The group’s distribution will not be disclosed unless the researchers had to know the details of the medication being taken by a participant. The study blind will be broken after all participants have finished the study. In case of emergency (such as serious adverse events, or suspected unexpected serious adverse reactions), or when the participant needs to be rescued and must know what kind of treatment the patient has received, the study blind will be opened after consultation of the administrator of research organization.

### Intervention

#### Conventional therapy

All enrolled subjects will receive conventional treatment based on the 2019 Chinese expert consensus for the diagnosis and treatment of AE-IPF [20] and 2018. An official ATS/ERS/JRS/ALAT statement [1]. So far, the pathogenesis of AE-IPF is not clearly understood, and it lacks effective treatment. The current clinical treatment measures mainly include (1) corticosteroids, (2) supplemental oxygen and noninvasive mechanical ventilation, and (3) symptomatic treatment.

#### TCM

The intervention group will be treated with Kangxianhuanji granule, and the control group will be given a placebo granule. Kangxianhuanji granule is a compound herbal medicine, and its main herbs are shown in Table 2. The Kangxianhuanji granule and the placebo granule come in bags, 10 g/bag, and were produced by Jiangyin TianJiang Pharmaceutical Co., Ltd. Each type of granule mixed in water for the oral taking, 10 g each time, twice a day for 4 weeks. Any Chinese herbal medicine or Chinese Patent Medicine with efficacy similar to the experimental drugs will be banned or restricted during the study.

**Table 2:** The main components of Kangxianhuanji granule

Chinese name	Latin name	English name	Amount (g)
Ren Shen	Ginseng Radix et Rhizoma	Ginseng	6
Huang Qi	Astragali Radix	Milkvetch root	20
Jiao Gu Lan	Herba GynoStemmae Pentaphilli	Fiveleaf gynostemma	15
Wu Wei Zi	Schisandrae Chinensis Fructus	Chinese magnoliavine fruit	9
Yin Yang Huo	Epimedii Folium	Short-horned Epimedium Herb	9

### Outcomes

#### Primary outcome

1. Treatment failure rate: The treatment failure is defined as the symptoms or signs that have not improved or worsened (intubation, treatment with a ventilator, etc.), or the occurrence of new significant symptoms and signs, or new infection, or death. The number of treatment failures in two groups was recorded during the treatment period (4 weeks).
2. All-cause mortality at 16 weeks (end of the follow-up): The number of deaths will be recorded at 16 weeks.

#### Secondary outcomes

1. Length of hospitalization: The length of hospitalization will be recorded during the treatment.
2. Overall Survival: Survival time up to 16 weeks.
3. Acute exacerbation rate: The number of participants with AE-IPF will be recorded during the follow-up period.
4. Intubation rate: The number of participants with intubation will be recorded throughout the study period.
5. Dyspnea: The Modified British Medical Research Council (mMRC) questionnaire [21] was used to assess dyspnea, as the mMRC reflects breathlessness in patients with IPF, and it could be used as a simple screening tool for efficacy assessment [22]. It will be completed and recorded at baseline, 7 days, 2 weeks, 4 weeks (end of the treatment), and 16 weeks (end of the follow-up). The mean change of the mMRC score between baseline to 4 weeks and 16 weeks will be statistically analyzed.
6. Quality of life: The IPF-specific version of the St George’s Respiratory Questionnaire for idiopathic pulmonary fibrosis (SGRQ-I) will be adopted to assess the health-related quality of life (HRQL) of patients with AE-IPF. The SGRQ-I was developed based on patients with IPF [23, 24]. It will be completed and recorded at baseline, 7 days, 2 weeks, 4 weeks (end of the treatment), and16 weeks (end of the follow-up). The mean change of the SGRQ-I total score between baseline to 4 weeks and 16 weeks will be statistically analyzed.
7. The partial pressure of oxygen (PaO_2_) and oxygen index: Arterial blood gas analysis will be tested and recorded at baseline, 7 days, and 2 weeks.

### Safety assessment

Any adverse event will be recorded for evaluating the safety of the Kangxianhuanji granule. Besides, routine blood tests, liver and kidney function, and electrocardiograph examinations will be performed before and after the treatment will be used to assess safety.

Any adverse event will be recorded in time, and the results of the causal assessment will be recorded. If a severe adverse event occurs in the study, it will be reported to the project manager and the Ethics Committee immediately. They will decide on whether to continue or stop the study. All adverse events should be tracked until properly resolved or stabilized.

After the study, the subjects should continue to be given necessary health guidance to tell them that they will still be concerned and will be followed up regularly. The researcher shall keep in touch with the subjects, instruct them to take medicine on time, and understand their diet, activities, rest, and medication. Timely give corresponding psychological counseling according to the subject's condition and psychological changes. In case of any change of condition, contact the doctor or go to the hospital for diagnosis and treatment in time.

### Data management and monitoring

Data collected in this trial will be derived from case report forms (CRFs). Any information about the primary and secondary outcomes at each timeline of the study will be recorded in the CRFs by the outcome assessors. The study monitors will be responsible for checking the integrity and quality of the completed CRFs. Two independent data administrators will be responsible for recording data on the Data Management System, which designed and built by Jiangsu Famous Medical Technology Co., Ltd. in Nanjing, China. The personal information of the participants will be protected, and the database can only be accessed with the permission of the research leader unit.

This trial refers to the universal standard of clinical trial audit [25], and the inspection mainly includes clinical trial project inspection and quality and risk management. The audit of the clinical trial project focuses on the authenticity and integrity of research data and is carried out in the preparation stage, the progress stage, and the conclusion stage of the clinical trial, respectively.

During the study, the following measures were taken to ensure the confidentiality and security of the subjects’ personal information. First, create a code to replace the recognizable information of the subject with an irrelevant character sequence. Secondly, use encrypted files and storage media that need to input passwords to ensure the maintenance and link of data in different systems. Third, limit the number of people who can carry out quality control, inspection, and analysis.

### Statistical analysis

The statistical analysis in this trial will be performed by Jiangsu Famous Medical Technology Co., Ltd. in Nanjing, China. The analysis will be based on the full analysis set, which is performed based on the ITT population.

In the descriptive data, continuous variables are described as mean (SD) and standard deviation or median and interquartile, categorical variables are described as numbers and percentages. The primary outcome of this study is treatment failure rate and all-cause mortality rate in each group. They will be compared between groups using the *χ*^2^ test or Fisher’s exact test. In secondary outcome analysis, the mMRC score for dyspnea, the SGRQ-I score for quality of life, the PaO_2_, and oxygen index will be used to analyze the difference, and differences between groups in mean change between baseline and measurements made at each time point will be analyzed by analysis of covariance. The length of hospitalization will be compared using the independent Student’s *t*-test or the Wilcoxon rank-sum test according to the normality of the data. Overall Survival, acute exacerbation rate, and intubation rate will be compared between groups using the *χ*^2^ test or Fisher’s exact test. The two groups will be compared using the 95% confidence interval method to determine superiority. SAS®, Version 9.2, performed all statistical analyses.

## Discussion

According to the clinical manifestations and characteristics of IPF, most scholars believe that IPF belongs to lung wilt disease or lung bi in TCM [26, 27]. There are specific effects and advantages in IPF treated by TCM. Previous studies have shown that TCM is beneficial in reducing symptoms such as cough, sputum, wheezing, and dyspnea, shortening the course of the disease and improving the quality of life [16, 28], but mostly focusing on the stable period. The research on the treatment of AE-IPF with TCM is still in the exploratory stage.

Western medicine is still currently a lack of effective treatment and control measures, and the treatment of AE-IPF only includes supportive care (i.e., palliation of symptoms and supplemental oxygen) and unproven interventions (such as corticosteroids, antibiotics, or lung transplantation) [29]. According to TCM, the primary pathogenesis of AE-IPF obstructs lung collaterals and phlegm and blood stasis. IPF patients experience external pathogenic factors due to the deficiency of the lung, abnormal lung qi circulation and descending, qi stagnation and blood stasis lead to lung collateral obstruction; stagnation of qi and blood stasis affect the healthy transfusion of water, and causing turbid phlegm to accumulate in the lung. The aggravation of phlegm and blood stasis leads to the occurrence of AE-IPF. The treatment of AE-IPF should be based on reinforcing the healthy qi and eliminating the pathogenic factors. Based on nourishing the kidney and lung, and treated by resolving phlegm, activating blood, and dredging collaterals.

Based on the core pathogenesis and treatment principles of AE-IPF, we have formulated Kangxianhuanji Granule, which was widely used in clinical practice and had an excellent curative effect. However, there are still no rigorously designed clinical trials to assess the efficacy of Kangxianhuanji Granule on AE-IPF. We applied for this exploratory clinical trial to estimate the security and feasibility of the Kangxianhuanji granule for AE-IPF. The results obtained from the study will provide evidence for the future design of high-quality, large-scale randomized controlled trials.

There are some limitations to this study. First, there is no proven, effective treatment for AE-IPF at present. High-dose corticosteroids and antibiotics are widely used, but the dose of appropriate and duration therapy remains unclear. It will result in a very different primary treatment for each participant. Another limitation is that due to the lack of preliminary evidence-based studies, the sample size estimated by exploratory research is too small, and the follow-up time is short. The results and conclusions will be limited. However, based on the result obtained from this study, a clinical trial with a long follow-up period and a large sample size will be conducted.

## Trial status

The recruitment of participants is in progress. Participant recruitment began on November 19, 2019, and will be completed on December 31, 2021.

## Data Availability

The study results will be available as the published manuscripts, and no additional unpublished data are available.

## Abbreviations

AE-IPF: Acute exacerbation of idiopathic pulmonary fibrosis
HRCT: High-resolution computed tomography
mMRC: Modified British Medical Research Council
SGRQ-I: St George’s Respiratory Questionnaire for idiopathic pulmonary fibrosis
HRQL: Health-related quality of life
PaO_2_: Partial pressure of oxygen
